# 2-Chloro-1-(4,5,6,7-tetrahydro­thieno[3,2-*c*]pyridin-5-yl)ethanone

**DOI:** 10.1107/S1600536810046805

**Published:** 2010-11-17

**Authors:** Chuan-Wei Yang, Miao Yang, Deng-Ke Liu, Ping-Bao Wang

**Affiliations:** a School of Pharmacy, Henan University, Henan, 475004, People’s Republic of China; b Tianjin Institute of Pharmaceutical Research, Tianjin, 300193, People’s Republic of China

## Abstract

In the title compound, C_9_H_10_ClNOS, the dihedral angle between the planar thio­phene ring and 2-chloro­ethane moiety (r.m.s deviations of 0.003 and 0.015 Å, respectively) is 45.79 (6)°. The tetra­hydro­pyridine ring adopts a half-chair conformation. The crystal packing reveals inter­molecular C—H⋯O inter­actions.

## Related literature

The title compound is an inter­mediate in the synthesis of thienopyridine compounds, which are characterized by anti­platelet activity. For background to thienopyridine derivatives, see: Kam & Nethery (2003 ▶). For bond-length data, see: Allen *et al.* (1987 ▶). For ring conformational analysis, see: Cremer & Pople (1975 ▶). For the preparation of 4,5,6,7-tetra­hydro-thieno[3,2-*c*]pyridine hydro­chloride, see: Lodewijk & Khatri (1989 ▶).
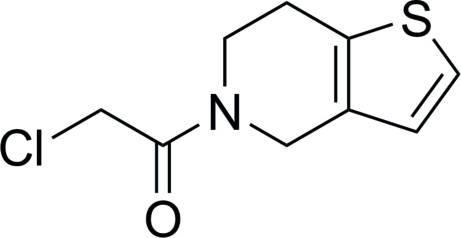


## Experimental

### 

#### Crystal data

C_9_H_10_ClNOS
*M* *_r_* = 215.69
Orthorhombic, 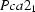

*a* = 10.5753 (4) Å
*b* = 10.8291 (4) Å
*c* = 8.0679 (3) Å
*V* = 923.94 (6) Å^3^
*Z* = 4
Mo *K*α radiation
μ = 0.59 mm^−1^
*T* = 113 K
0.26 × 0.24 × 0.18 mm

#### Data collection

Rigaku Saturn CCD area-detector diffractometer
Absorption correction: multi-scan (*CrystalClear*; Rigaku/MSC, 2005 ▶) *T*_min_ = 0.861, *T*_max_ = 0.901
8326 measured reflections
1927 independent reflections
1850 reflections with *I* > 2σ(*I*)
*R*_int_ = 0.026

#### Refinement

*R*[*F* ^2^ > 2σ(*F* ^2^)] = 0.020
*wR*(*F* ^2^) = 0.057
*S* = 1.09
1927 reflections
119 parameters
1 restraint
H-atom parameters constrained
Δρ_max_ = 0.23 e Å^−3^
Δρ_min_ = −0.20 e Å^−3^
Absolute structure: Flack (1983 ▶), 755 Friedel pairs
Flack parameter: 0.02 (5)

### 

Data collection: *CrystalClear* (Rigaku/MSC, 2005 ▶); cell refinement: *CrystalClear*; data reduction: *CrystalClear*; program(s) used to solve structure: *SHELXS97* (Sheldrick, 2008 ▶); program(s) used to refine structure: *SHELXL97* (Sheldrick, 2008 ▶); molecular graphics: *SHELXTL* (Sheldrick, 2008 ▶); software used to prepare material for publication: *CrystalStructure* (Rigaku/MSC, 2005 ▶) and *PLATON* (Spek, 2009 ▶).

## Supplementary Material

Crystal structure: contains datablocks global, I. DOI: 10.1107/S1600536810046805/kp2289sup1.cif

Structure factors: contains datablocks I. DOI: 10.1107/S1600536810046805/kp2289Isup2.hkl

Additional supplementary materials:  crystallographic information; 3D view; checkCIF report

## Figures and Tables

**Table 1:** Hydrogen-bond geometry (Å, °)

*D*—H⋯*A*	*D*—H	H⋯*A*	*D*⋯*A*	*D*—H⋯*A*
C9—H9*A*⋯O1^i^	0.99	2.55	3.356 (2)	138

Symmetry code: (i) 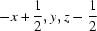.

**Fig. 1.**
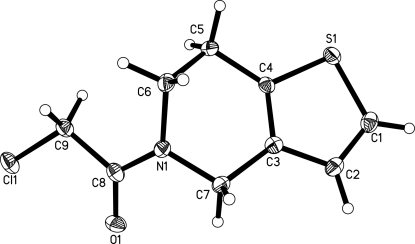
The molecular structure of (I), Displacement ellipsoids are drawn at the 50% probability level.

## supplementary crystallographic information

### Comment

The thienopyridines ticlopidine and clopidogrel are inhibitors of platelet function in vivo. They are effective in preventing atherothrombotic events in cardiovascular, cerebrovascular, and peripheral vascular disease (Kam & Nethery, 2003). The crystal structure of the title compound, 2-chloro-1-(6,7-dihydrothieno [3,2-*c*]pyridin-5(4*H*)-yl)ethanone (I), an intermediate in the synthesis of some of the thienopyridines, is reported here.

In the title compound (Fig. 1) all bond lengths and angles in (I) are within normal ranges (Allen *et al.*, 1987). The thiophene ring is planar (r.m.s. deviation 0.003 Å for  C8/C9/N1/O1/Cl1). The half chair conformation of the tetrahydropyridine ring is defined by the puckering parameter of φ_2_=217.5 (2) ° and QT=0.5052 (15) Å (Cremer & Pople, 1975). The packing is realised by intermolecular C—H···O (Table 1) interactions.

### Experimental

The synthesis of 4,5,6,7-tetrahydro-thieno[3,2-*c*]pyridine hydrochloride was reported by Lodewijk & Khatri (1989). In our experiment, 4,5,6,7-tetrahydro-thieno[3,2-*c*]pyridine was released from 4,5,6,7-tetrahydrothieno[3,2,c]pyridine hydrochloride (5.0 g, 29 mmol) by reaction with NaHCO_3_ (7.3 g, 87 mmol) in the presence of CH_2_Cl_2_ (50 mL) and water (15 mL), stirred for 4 h at 273 K. The organic phase was washed with water and evaporated off under reduced pressure to get yellow oil (3.9 g, 28 mmol). The oil was dissolved in CH_2_Cl_2_ (50 mL) and 2-chloroacetyl chloride (3.5 g, 31 mmol) was added dropwise into the mixture. The mixture was stirred at 268 K for 5 h, the organic phase was washed with water and evaporated off under reduced pressure to get yellow oil (5.7 g) as a crude product. The product was dissolved in a mixture of petroleum ether (40 mL) and acetone (10 mL) at 273 K, and white crystals were grown slowly.

### Refinement

All the H atoms was located on their parent atoms with C—H=0.95Å (aromatic CH) and 0.99Å (CH2), *U*_iso_ = 1.2*U*_eq_(C).

### Crystal data

**Table:**

C_9_H_10_ClNOS	*F*(000) = 448
*M**_r_* = 215.69	*D*_x_ = 1.551 Mg m^−^^3^
Orthorhombic, *P**c**a*2_1_	Mo *K*α radiation, λ = 0.71070 Å
Hall symbol: P 2c -2ac	Cell parameters from 3069 reflections
*a* = 10.5753 (4) Å	θ = 1.9–27.9°
*b* = 10.8291 (4) Å	µ = 0.59 mm^−^^1^
*c* = 8.0679 (3) Å	*T* = 113 K
*V* = 923.94 (6) Å^3^	Block, colourless
*Z* = 4	0.26 × 0.24 × 0.18 mm

### Data collection

**Table:**

Rigaku Saturn CCD area detector diffractometer	1927 independent reflections
Radiation source: rotating anode	1850 reflections with *I* > 2σ(*I*)
confocal	*R*_int_ = 0.026
Detector resolution: 7.31 pixels mm^-1^	θ_max_ = 27.9°, θ_min_ = 1.9°
ω and φ scans	*h* = −13→13
Absorption correction: multi-scan *CrystalClear*	*k* = −14→14
*T*_min_ = 0.861, *T*_max_ = 0.901	*l* = −10→8
8326 measured reflections	

### Refinement

**Table:**

Refinement on *F*^2^	Hydrogen site location: inferred from neighbouring sites
Least-squares matrix: full	H-atom parameters constrained
*R*[*F*^2^ > 2σ(*F*^2^)] = 0.020	*w* = 1/[σ^2^(*F*_o_^2^) + (0.0393*P*)^2^] where *P* = (*F*_o_^2^ + 2*F*_c_^2^)/3
*wR*(*F*^2^) = 0.057	(Δ/σ)_max_ = 0.001
*S* = 1.09	Δρ_max_ = 0.23 e Å^−^^3^
1927 reflections	Δρ_min_ = −0.20 e Å^−^^3^
119 parameters	Extinction correction: *SHELXL*, Fc^*^=kFc[1+0.001xFc^2^λ^3^/sin(2θ)]^-1/4^
1 restraint	Extinction coefficient: 0.035 (5)
Primary atom site location: structure-invariant direct methods	Absolute structure: Flack (1983), 743 Friedel pairs
Secondary atom site location: difference Fourier map	Flack parameter: 0.02 (5)

### Special details

**Table:**

Geometry. All e.s.d.'s (except the e.s.d. in the dihedral angle between two l.s. planes) are estimated using the full covariance matrix. The cell e.s.d.'s are taken into account individually in the estimation of e.s.d.'s in distances, angles and torsion angles; correlations between e.s.d.'s in cell parameters are only used when they are defined by crystal symmetry. An approximate (isotropic) treatment of cell e.s.d.'s is used for estimating e.s.d.'s involving l.s. planes.
Refinement. Refinement of *F*^2^ against ALL reflections. The weighted *R*-factor *wR* and goodness of fit *S* are based on *F*^2^, conventional *R*-factors *R* are based on *F*, with *F* set to zero for negative *F*^2^. The threshold expression of *F*^2^ > σ(*F*^2^) is used only for calculating *R*-factors(gt) *etc*. and is not relevant to the choice of reflections for refinement. *R*-factors based on *F*^2^ are statistically about twice as large as those based on *F*, and *R*- factors based on ALL data will be even larger.

### Fractional atomic coordinates and isotropic or equivalent isotropic displacement parameters (Å^2^)

**Table:**

	*x*	*y*	*z*	*U*_iso_*/*U*_eq_	
Cl1	0.26658 (4)	−0.10564 (3)	0.20010 (5)	0.02247 (11)	
S1	0.01605 (3)	0.55872 (3)	0.34620 (6)	0.01748 (10)	
O1	0.35771 (11)	0.10076 (10)	0.39637 (14)	0.0196 (3)	
N1	0.17029 (11)	0.18603 (10)	0.47077 (16)	0.0144 (3)	
C1	0.14078 (15)	0.62110 (13)	0.4538 (2)	0.0186 (3)	
H1	0.1563	0.7072	0.4633	0.022*	
C2	0.21493 (14)	0.53226 (13)	0.52392 (19)	0.0171 (3)	
H2	0.2877	0.5491	0.5891	0.021*	
C3	0.17003 (14)	0.41084 (12)	0.48778 (19)	0.0141 (3)	
C4	0.06333 (14)	0.40982 (12)	0.39463 (19)	0.0136 (3)	
C5	−0.01059 (13)	0.29663 (11)	0.3500 (3)	0.0162 (3)	
H5A	0.0058	0.2734	0.2333	0.019*	
H5B	−0.1023	0.3123	0.3631	0.019*	
C6	0.03142 (13)	0.19294 (12)	0.4661 (2)	0.0160 (3)	
H6A	−0.0015	0.2086	0.5790	0.019*	
H6B	−0.0035	0.1133	0.4269	0.019*	
C7	0.22995 (14)	0.29303 (12)	0.5499 (2)	0.0161 (3)	
H7A	0.3215	0.2936	0.5246	0.019*	
H7B	0.2198	0.2873	0.6717	0.019*	
C8	0.24161 (16)	0.10101 (12)	0.3917 (2)	0.0156 (3)	
C9	0.16586 (13)	0.00648 (13)	0.29136 (19)	0.0171 (3)	
H9A	0.1180	0.0496	0.2032	0.021*	
H9B	0.1043	−0.0352	0.3649	0.021*	

### Atomic displacement parameters (Å^2^)

**Table:**

	*U*^11^	*U*^22^	*U*^33^	*U*^12^	*U*^13^	*U*^23^
Cl1	0.0279 (2)	0.01909 (17)	0.02041 (19)	0.00927 (14)	−0.00056 (18)	−0.00199 (16)
S1	0.01631 (18)	0.01532 (16)	0.02081 (19)	0.00237 (13)	−0.00046 (18)	0.00111 (16)
O1	0.0134 (6)	0.0224 (5)	0.0229 (6)	0.0025 (4)	0.0001 (4)	0.0021 (4)
N1	0.0134 (6)	0.0131 (5)	0.0167 (6)	−0.0018 (4)	0.0003 (5)	0.0000 (5)
C1	0.0198 (8)	0.0159 (6)	0.0201 (8)	−0.0039 (5)	0.0037 (6)	−0.0013 (6)
C2	0.0161 (8)	0.0189 (7)	0.0163 (8)	−0.0033 (5)	0.0011 (6)	−0.0025 (6)
C3	0.0130 (7)	0.0162 (6)	0.0131 (7)	−0.0011 (5)	0.0024 (6)	0.0005 (5)
C4	0.0114 (7)	0.0138 (6)	0.0157 (8)	0.0014 (5)	0.0033 (5)	−0.0002 (5)
C5	0.0116 (6)	0.0170 (6)	0.0198 (7)	−0.0001 (5)	−0.0018 (6)	−0.0029 (7)
C6	0.0127 (7)	0.0140 (6)	0.0214 (8)	−0.0023 (5)	0.0043 (6)	−0.0021 (6)
C7	0.0155 (7)	0.0150 (6)	0.0179 (8)	−0.0019 (5)	−0.0036 (6)	0.0002 (6)
C8	0.0193 (8)	0.0146 (6)	0.0128 (7)	0.0020 (5)	0.0005 (6)	0.0049 (5)
C9	0.0173 (7)	0.0165 (6)	0.0175 (7)	0.0043 (5)	0.0001 (6)	−0.0016 (5)

### Geometric parameters (Å, °)

**Table:**

Cl1—C9	1.7751 (14)	C3—C7	1.5103 (18)
S1—C1	1.7173 (17)	C4—C5	1.4977 (18)
S1—C4	1.7329 (14)	C5—C6	1.528 (2)
O1—C8	1.2283 (19)	C5—H5A	0.9900
N1—C8	1.3503 (19)	C5—H5B	0.9900
N1—C7	1.4658 (17)	C6—H6A	0.9900
N1—C6	1.4710 (18)	C6—H6B	0.9900
C1—C2	1.364 (2)	C7—H7A	0.9900
C1—H1	0.9500	C7—H7B	0.9900
C2—C3	1.4280 (19)	C8—C9	1.532 (2)
C2—H2	0.9500	C9—H9A	0.9900
C3—C4	1.356 (2)	C9—H9B	0.9900
			
C1—S1—C4	91.75 (7)	N1—C6—C5	110.08 (11)
C8—N1—C7	120.29 (12)	N1—C6—H6A	109.6
C8—N1—C6	125.47 (13)	C5—C6—H6A	109.6
C7—N1—C6	113.62 (11)	N1—C6—H6B	109.6
C2—C1—S1	111.95 (11)	C5—C6—H6B	109.6
C2—C1—H1	124.0	H6A—C6—H6B	108.2
S1—C1—H1	124.0	N1—C7—C3	110.02 (12)
C1—C2—C3	111.93 (13)	N1—C7—H7A	109.7
C1—C2—H2	124.0	C3—C7—H7A	109.7
C3—C2—H2	124.0	N1—C7—H7B	109.7
C4—C3—C2	113.42 (13)	C3—C7—H7B	109.7
C4—C3—C7	121.76 (12)	H7A—C7—H7B	108.2
C2—C3—C7	124.78 (13)	O1—C8—N1	123.06 (14)
C3—C4—C5	125.05 (13)	O1—C8—C9	122.51 (13)
C3—C4—S1	110.95 (10)	N1—C8—C9	114.41 (13)
C5—C4—S1	123.83 (11)	C8—C9—Cl1	111.26 (10)
C4—C5—C6	107.60 (13)	C8—C9—H9A	109.4
C4—C5—H5A	110.2	Cl1—C9—H9A	109.4
C6—C5—H5A	110.2	C8—C9—H9B	109.4
C4—C5—H5B	110.2	Cl1—C9—H9B	109.4
C6—C5—H5B	110.2	H9A—C9—H9B	108.0
H5A—C5—H5B	108.5		
			
C4—S1—C1—C2	−0.19 (13)	C7—N1—C6—C5	67.73 (16)
S1—C1—C2—C3	0.67 (18)	C4—C5—C6—N1	−48.83 (16)
C1—C2—C3—C4	−1.0 (2)	C8—N1—C7—C3	126.36 (14)
C1—C2—C3—C7	−178.84 (14)	C6—N1—C7—C3	−45.10 (16)
C2—C3—C4—C5	−174.63 (15)	C4—C3—C7—N1	10.1 (2)
C7—C3—C4—C5	3.3 (2)	C2—C3—C7—N1	−172.22 (13)
C2—C3—C4—S1	0.80 (17)	C7—N1—C8—O1	7.9 (2)
C7—C3—C4—S1	178.75 (12)	C6—N1—C8—O1	178.27 (14)
C1—S1—C4—C3	−0.36 (13)	C7—N1—C8—C9	−170.49 (12)
C1—S1—C4—C5	175.15 (15)	C6—N1—C8—C9	−0.1 (2)
C3—C4—C5—C6	16.2 (2)	O1—C8—C9—Cl1	4.40 (19)
S1—C4—C5—C6	−158.65 (11)	N1—C8—C9—Cl1	−177.22 (11)
C8—N1—C6—C5	−103.21 (16)		

### Hydrogen-bond geometry (Å, °)

**Table:**

*D*—H···*A*	*D*—H	H···*A*	*D*···*A*	*D*—H···*A*
C9—H9A···O1^i^	0.99	2.55	3.356 (2)	138

Symmetry codes: (i) −*x*+1/2, *y*, *z*−1/2.
